# Involvement of the capsular GalXM-induced IL-17 cytokine in the control of *Cryptococcus neoformans* infection

**DOI:** 10.1038/s41598-018-34649-4

**Published:** 2018-11-06

**Authors:** Isabel Ferreira LaRocque-de-Freitas, Juliana Dutra B. Rocha, Marise Pinheiro Nunes, Priscila Angelica V. Oliveira, Danielle de Oliveira Nascimento, Leonardo Freire-de-Lima, Christina Maeda Takiya, Alexandre Morrot, Debora Decote-Ricardo, Jose Osvaldo Previato, George A. DosReis, Lucia Mendonça-Previato, Celio Geraldo Freire-de-Lima

**Affiliations:** 1 Instituto de Biofísica Carlos Chagas Filho, Universidade Federal do Rio de Janeiro, Rio de Janeiro, 21941-900 Brazil; 2 Laboratório de Imunoparasitologia, Instituto Oswaldo Cruz, FIOCRUZ, Rio de Janeiro, 21045-900 Brazil; 3 Faculdade de Medicina, Universidade Federal do Rio de Janeiro, Rio de Janeiro, 21941-900 Brazil; 4 Instituto de Veterinária, Universidade Federal Rural do Rio de Janeiro, Seropédica, 23890-000 Brazil

## Abstract

*Cryptococcus neoformans* is an opportunistic fungus that can cause lethal brain infections in immunosuppressed individuals. Infection usually occurs via the inhalation of a spore or desiccated yeast which can then disseminate from the lung to the brain and other tissues. Dissemination and disease is largely influence by the production of copious amounts of cryptococcal polysaccharides, both which are secreted to the extracellular environment or assembled into a thick capsule surrounding the cell body. There are two important polysaccharides: glucuronoxylomannan (GXM) and galactoxylomannan, also called as glucuronoxylomanogalactan (GXMGal or GalXM). Although GXM is more abundant, GalXM has a more potent modulatory effect. In the present study, we show that GalXM is a potent activator of murine dendritic cells, and when co-cultured with T cells, induces a Th17 cytokine response. We also demonstrated that treating mice with GalXM prior to infection with *C. neoformans* protects from infection, and this phenomenon is dependent on IL-6 and IL-17. These findings help us understand the immune biology of capsular polysaccharides in fungal pathogenesis.

## Introduction

*Cryptococcus neoformans* is an environmental yeast that has a polysaccharide capsule and can cause meningoencephalitis in immunosuppressed hosts and eventually, in immunocompetent individuals^1–3^. Cryptococcosis begins when the individual inhales the sporulated form of *C. neoformans* present in the environment. The microorganisms from the lung spread through the bloodstream to reach different vertebrate^4–6^ host organs, after which they can invade the CNS^7–10^. Persistence and dissemination in the host is largely influenced by Cryptococcal polysaccharides, which are both secreted or assembled into a think polysaccharide capsule. The capsule consists primarily of 88% glucuronoxylomannan (GXM). GXM is a polymer that consists mostly of an α-(1–3)-mannan substituted with β-(1–2)-glucopyranosyluronic acid and β-(1–4)-xylopyranosyl. O-acetylation occurs on the C-6 of about half of the mannose residues^11–15^. The *C. neoformans* capsule also contains 10% galactoxylomannan (GalXM) and 2% mannoproteins^16^. Galactoxylomannan consists of an α-(1 → 6)-galactan backbone with galactomannan side chains that are further substituted with variable numbers of xylose and glucuronic acid residues^16–19^.

These two capsular polysaccharides can act on the immune system in different ways. GXM has already been characterized as a molecule with immunosuppressive activity on monocytes/macrophages, neutrophils, and dendritic cells. Monocytes/macrophages are involved in the capture and internalization of GXM mediated by Toll-like receptors, CD14, CD18, and the IgG receptor FcgRIIB^20–27^.

Retini and colleagues^28^ found that GXM blocked the production of interleukin (IL)-12 by monocytes and increased the secretion of IL-10 when stimulated monocytes were co-cultured with T cells^28^. In addition, GXM induced transforming growth factor (TGF)-β in the macrophage cell line RAW 264.7^29^. Mice infected with encapsulated strains were unable to induce T-helper (Th) 1 cytokines such as IL-2 and interferon (IFN)-γ, inducing a significant accumulation of IL-10 that was not observed in the mice infected with an acapsular mutant. These results suggest that yeasts containing GXM on their surface limit the development of a Th1-type protective response in an inhibitory process in which IL-10 plays a critical role^28,30,31^. Our group recently showed that GXM does not induce the release of neutrophil extracellular traps (NETs) by human neutrophils and that in the presence of GXM, stimulated human neutrophils block NET release^32^. In addition to these immunomodulations, GXM can also induce apoptosis in different systems. Monari and colleagues^33^ demonstrated that FasL expression in murine macrophages induces apoptosis in activated T cells through processes involving intrinsic and extrinsic pathways^24,33,34^. It has also been shown that GXM can induce apoptosis in macrophages through a mechanism that involves an increase in Fas and FasL^29^.

The majority of studies on the immunomodulatory effects of capsular polysaccharides from *C. neoformans* have been performed with GXM, but the possible roles of GalXM as an immunomodulatory molecule remain unclear. Reports have increased in recent years suggesting this polysaccharide may also have important immunomodulatory activities. Chaka and colleagues^35^ showed that GalXM could induce the production of tumor necrosis factor (TNF)-α in peripheral blood mononuclear cells (PBMCs)^35^. The production of nitric oxide through the expression of inducible nitric oxide synthase and the release of TNF-α induced by GalXM have also been described^29^. Unlike the action of GXM on the production of NETs, Rocha and colleagues^32^ have shown that stimulation with GalXM or with acapsular fungus CAP67 (which lacks GXM in the polysaccharide capsule) is sufficient for the induction of NETs by human neutrophils^32^.

These observations suggest GXM and GalXM have different immunomodulatory activities. In addition, GalXM can induce apoptosis in different cells of the immune system. Pericolini and colleagues^36^ showed that GalXM induced apoptosis in memory T cells in rheumatoid arthritis patients^36^. Villena and colleagues^29^ also demonstrated that GalXM’s induction of apoptosis in the RAW cell line was mediated by Fas/FasL interactions, and the effect was ~50 times greater than that observed for GXM^29^. GalXM-mediated cell death might enhance the suppressive effect of GXM and contribute to suppression during cryptococcosis^37–39^. Furthermore, Moyrand *et al*. demonstrated the importance of GalXM in the virulence of *C. neoformans* by compromising its biosynthesis via UDP-Glc epimerase (uge1Δ) and UDP-Gal transporter (ugt1Δ)^40^.

In this report, we provide the first description of the *in vitro* modulation of dendritic cell activation and evaluate the participation of purified capsular polysaccharides. Our results showed that GalXM induced the production of IL-6, TGF-β, and IL-17 in co-cultures of stimulated dendritic cells and T lymphocytes, a characteristic profile for a Th17 response. The GalXM also induced a protective effect *in vivo* where the number of colony-forming units (CFUs) showed reductions in the fungal loads in different organs. Our data also show that GalXM induces a protective response in mice infected with *C. neoformans*, suggesting an effect mediated by Th17 lymphocytes during the early stages of infection.

## Results

### Capsular constituents GXM and GalXM induce the maturation of murine dendritic cells and the production of cytokines

**Figure 1:**
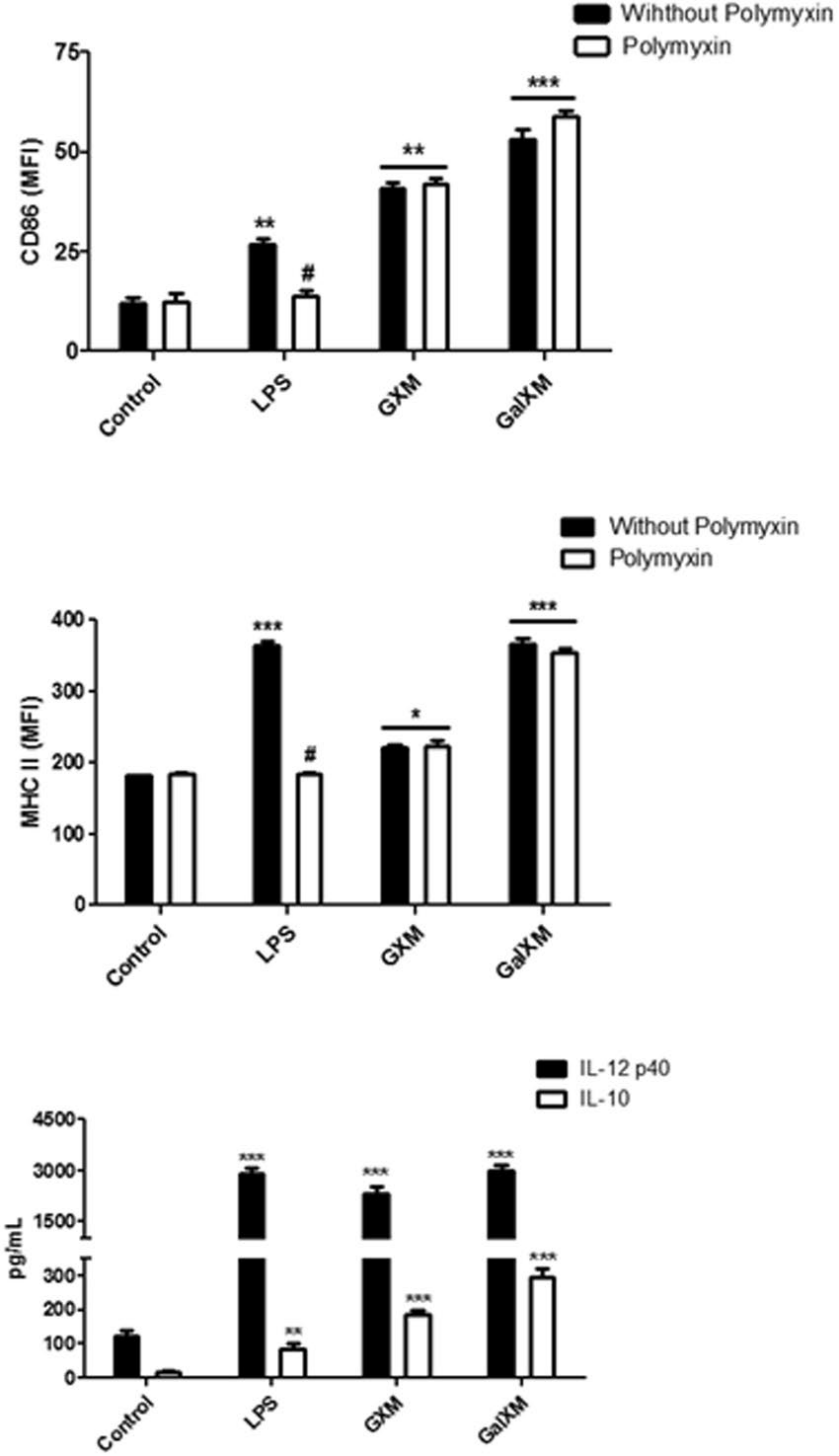
CD86 and MHC II expression and cytokine production in murine dendritic cells stimulated with GXM or GalXM capsular polysaccharides. Dendritic cells (2 × 10^5^/well) were incubated for 24 h in the presence of 50 μg/mL of capsular GXM or GalXM (wild-type strain B3501) from *Cryptococcus neoformans* or 10-ng/mL lipopolysaccharide (LPS) in the presence or absence of polymyxin B (100 ng/mL). Cells with labeled (**A**) CD11c (phycoerythrin; PE) and CD86 (FITC) or (**B**) CD11c (PE) and MHC II (FITC) were checked by flow cytometry along with positive cells. The control corresponds to dendritic cells without stimulus. The graph represents the mean fluorescence intensity (MFI). The result represents one of three independent experiments. The asterisks indicate ***P < 0.001, **P < 0.01, and *P < 0.05 compared to the control, and the hash symbol (#) indicates P < 0.01 compared to LPS without the addition of polymyxin B. To test cytokine production after stimulation, the culture supernatants were collected, and the production of cytokines IL-12p40 and IL-10 were determined with an ELISA assay. The control corresponds to dendritic cells without stimulus. The results represent one of four independent experiments. The asterisks indicate ***P < 0.001 and **P < 0.01 in relation to the control.

Dendritic cells are professional antigen-presenting cells in which T cells can initiate the primary and secondary responses^41–43^. Immature dendritic cells reside inside non-lymphoid organs where they actively capture and process antigens. Their maturation can be induced by contact with proinflammatory cytokines and by the recognition of microbial products migrating to the lymphoid organs, where the initiation of the adaptive immune response occurs^44^. To evaluate the involvement of the purified capsular constituents from *C. neoformans* on dendritic-cell maturation, the cells were incubated for 24 h in the presence or absence of 50-μg/mL GXM or GalXM. Both capsular constituents induced the increased expression of major histocompatibility complex class II (MHC II) and CD86 (surface structures characteristic of mature dendritic cells), with GalXM eliciting a more intense increase in marker expression than GXM (Fig. 1A,B). To eliminate potential LPS contamination of the polysaccharides, the experiments were performed with polymyxin B (Fig. 1A,B).

**Figure 2:**
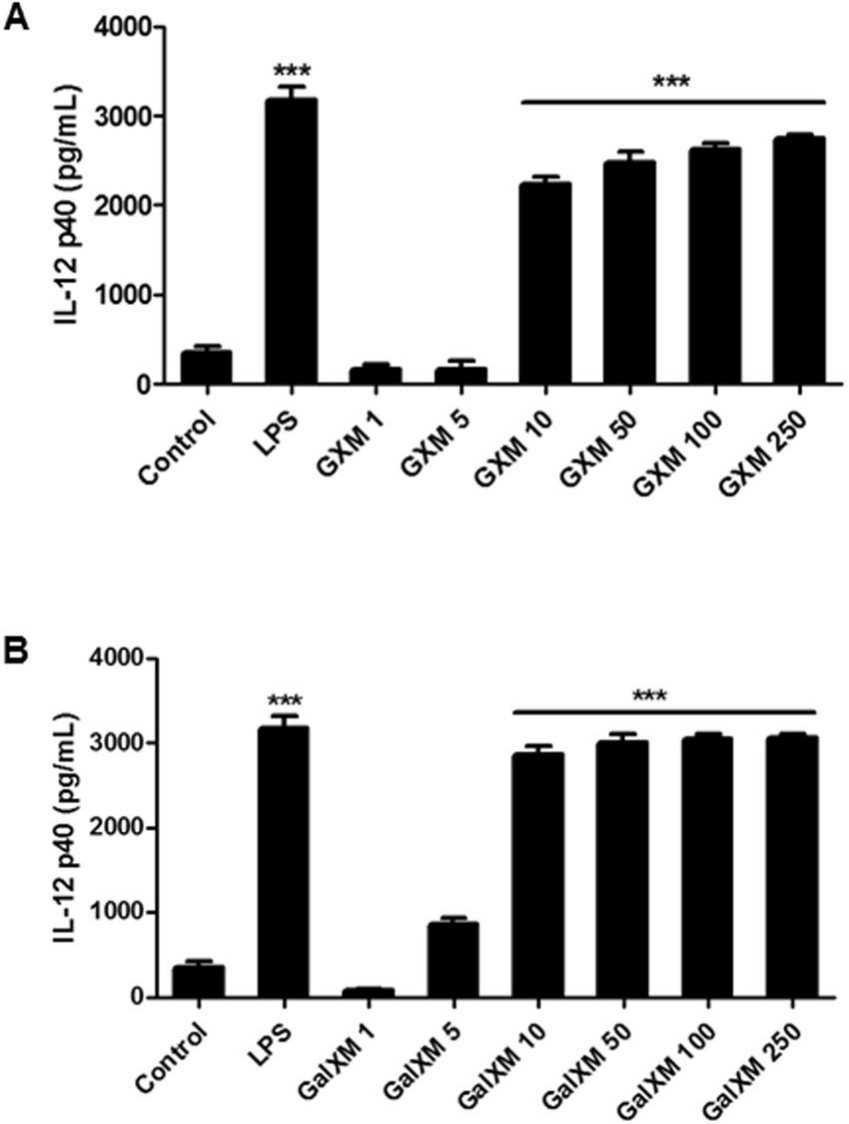
The effect of different concentrations of GXM and GalXM on the production of IL-12p40 by dendritic cells. Dendritic cells (2 × 10^5^/well) were incubated in the presence of different concentrations (μg/mL) of (**A**) GXM or (**B**) GalXM for 24 hours. The culture supernatants were then collected, and ELISA assays were used to determine the amount of the cytokine IL-12p40. The control (100 ng/mL LPS) corresponds to dendritic cells without stimulus. The results represent one of four independent experiments. The asterisks indicate ***P < 0.001 in relation to the control.

We also evaluated the cytokine profile produced by dendritic cells stimulated by the capsular constituents purified from *C. neoformans*. The results showed that both capsular polysaccharides (GXM and GalXM) induced a significant increase in the production of IL-10 (Fig. 1C). Furthermore, both capsular polysaccharides induced increased IL-12p40 cytokine production in the dendritic cells. Figure 2 shows the production of IL-12p40 at different concentrations of GXM and GalXM. Although some authors have used higher than 50 μg/mL concentrations^45,46^, our data show that small concentrations of GXM or GalXM (Fig. 2) result in the same level of IL-12p40 production as when larger concentrations (up to 250 μg/mL) are used. These experiments showed that the 50 μg/mL dosage did not compromise the results obtained and promoted the increase in IL-12p40 production indicative of dendritic cell activation.

### Increased lymphoproliferative response induced by GXM or GalXM capsule constituents

**Figure 3:**
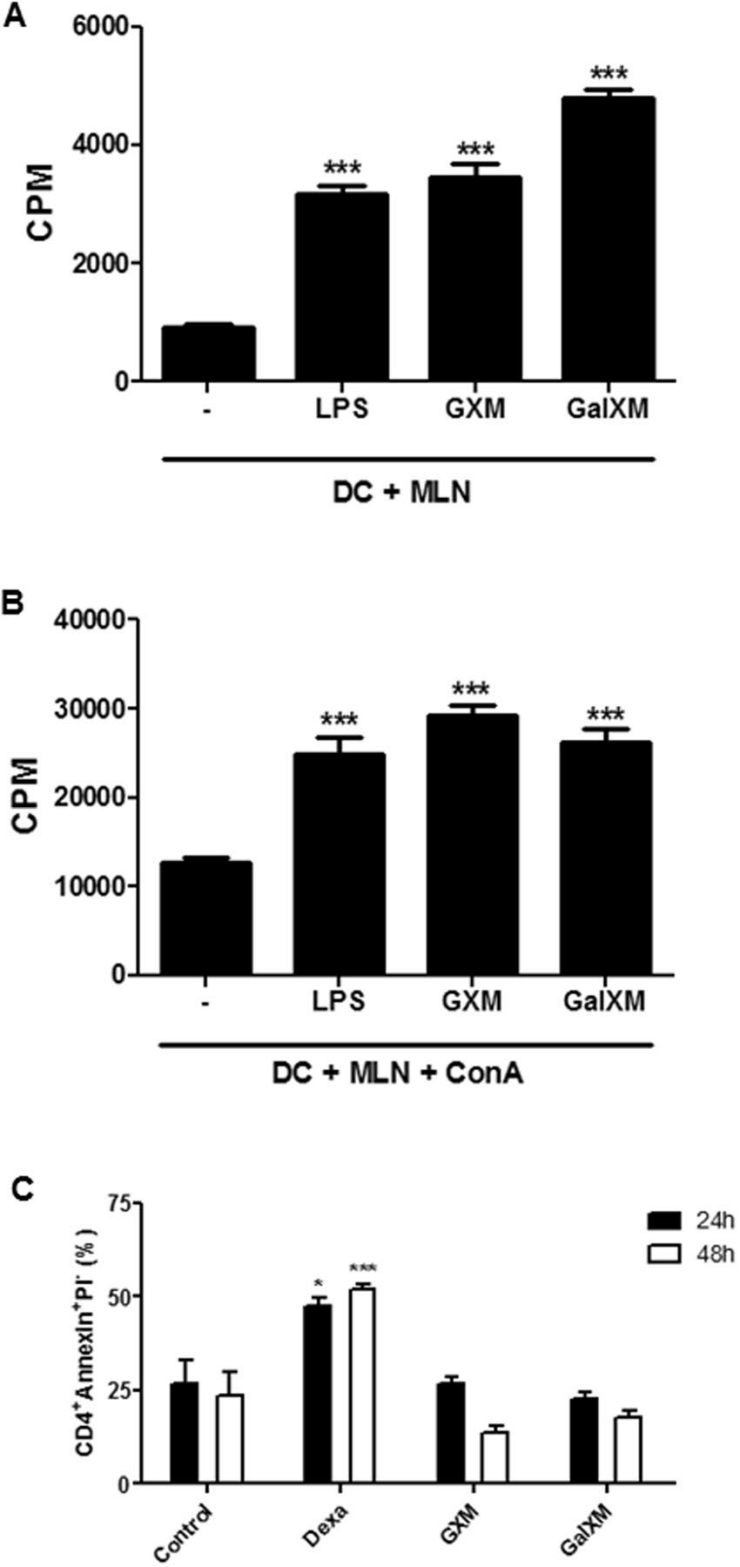
Increased *in vitro* lymphoproliferative response induced by murine dendritic cells treated with GXM or GalXM. Dendritic cells (3 × 10^4^/well) were incubated for 24 h in the presence of 50 μg/mL of the constituents’ capsule of *C. neoformans*. The dendritic cells were then incubated with (**A**) MLN cells (3 × 10^5^/well) or with (**B**) MLN (3 × 10^5^/well) in the presence of ConA (5 μg/mL). After 72 h, lymphoproliferation was analyzed by the incorporation of 3H-thymidine in the last 18 h of culture. Lipopolysaccharide (LPS) was used at a concentration of 100 ng/mL. (**C**) Dendritic cells were incubated with MLN cells (3 × 10^5^/well). After 24 or 48 hours, T cells were labeled with anti-CD4 (allophycocyanin) and the percentage of apoptotic cells was quantified by flow cytometry using annexin V (FITC) and propidium iodide. Dexamethasone (1 μM) was used as a positive control for apoptosis. The results represent one of three independent experiments. The asterisks indicate ***P < 0.001 and *P < 0.05 relative to the control.

Because the capsular constituents were able to induce the maturation of dendritic cells, we evaluated the effect of these GXM- or GalXM-stimulated cells on lymphocyte activation *in vitro*. The proliferative response of the total mesenteric lymph node (MLN) cells was significantly increased (Fig. 3B). When the MLN cells were exposed to ConA (5 μg/mL), the proliferative response was even higher (Fig. 3B).

As previously demonstrated by Pericolini and colleagues^37^, when interacting directly with human T lymphocytes and the Jurkat lineage, GalXM induces apoptosis via the activation of caspase 8 in the cells. We performed the annexin V/propidium iodide apoptosis assay with flow cytometry to detect phosphatidylserine expression on the cell surface. Neither the GXM- nor GalXM-activated dendritic cells induced cell death by apoptosis in CD4^+^ T cells when co-incubated for 24 or 48 hours (Fig. 3C).

We also evaluated whether capsular GXM or GalXM interacting with MLN cells could induce an increase in the proliferative response in the absence of dendritic cells or induce apoptosis as previously published by Pericolini and colleagues^37^. The capsular GXM did not induce a lymphoproliferative response directly, a result in agreement with Yauch and colleagues^46^. Moreover, GalXM did induce an increase in the lymphoproliferative response at concentrations of 10 or 50 μg/mL (Supplementary Fig. S1**)**, as previously described by Pericolini and colleagues^37^. These results show that the capsular constituents have different mechanisms of action, but acting together they may lead to a suppression of the immune response^29,37^.

### Production of IFN-γ by CD4^+^ T cells in cultures with murine dendritic cells pre-treated with GalXM

**Figure 4:**
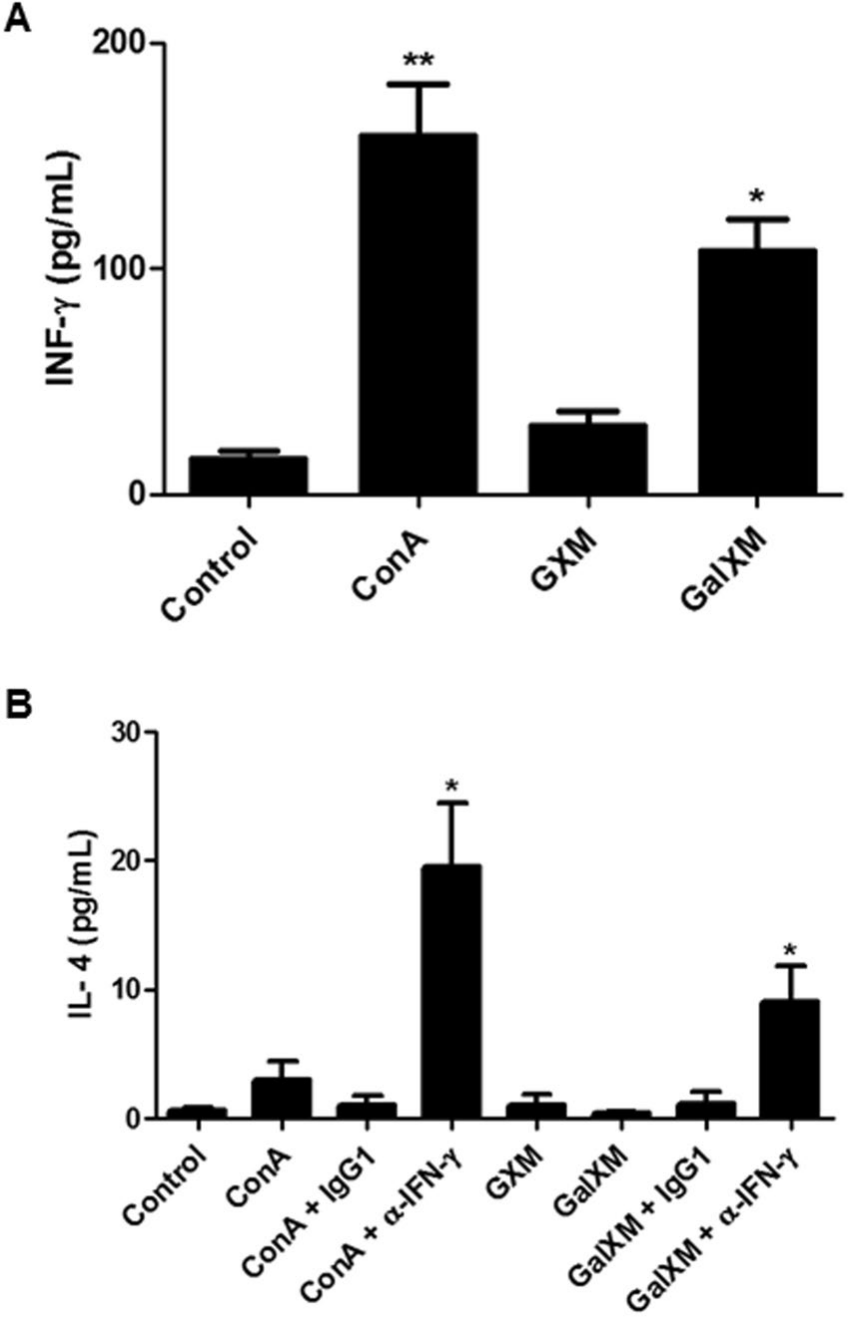
CD4^+^ T cells in culture with murine dendritic cells pretreated with GalXM secrete IFN-γ. Dendritic cells (3 × 10^4^/well) were incubated for 24 h in the presence of 50 μg/mL of the constituents’ capsule of *C. neoformans*. The dendritic cells were then incubated with CD4^+^ T cells (3 × 10^5^/well) for 48 h. The culture supernatant was collected, and ELISA assays were used to determine the production of (**A**) IFN-γ and (**B**) IL-4. ConA was used at 5 μg/mL, and anti-IFN-γ and its IgG1a κ control were used at a concentration of 3 μg/mL. The control corresponds to dendritic cells without stimulus. The results represent one of four independent experiments. The asterisks indicate **P < 0.01 and *P < 0.05 relative to the control.

As the MLN cells showed an impressive lymphoproliferative response when co-cultured with dendritic cells previously stimulated with both capsular constituents, we decided to evaluate the possible implications for the differentiation of T-cell subpopulations. CD4^+^ T cells were purified by negative selection to produce 92.14% CD4^+^-positive cells (data not shown). Dendritic cells stimulated with GXM co-cultivated with the CD4^+^ T cells were unable to induce differentiation to the Th1 or Th2 subpopulations, represented respectively by the cytokines IFN-γ and IL-4 (Fig. 4A,B). However, when dendritic cells stimulated with GalXM were co-cultured with T lymphocytes, a significant increase in IFN-γ production was observed (Fig. 4A). This result is in agreement with some previous reports, where it was demonstrated that during a fungal infection, patients develop a Th1-type response with CD4^+^ and CD8^+^ T-cell recruitment^47–51^.

### Dendritic cells stimulated with the capsular polysaccharide GalXM in co-culture with CD4^+^ T cells induced differentiation to the Th17 subtype

**Figure 5:**
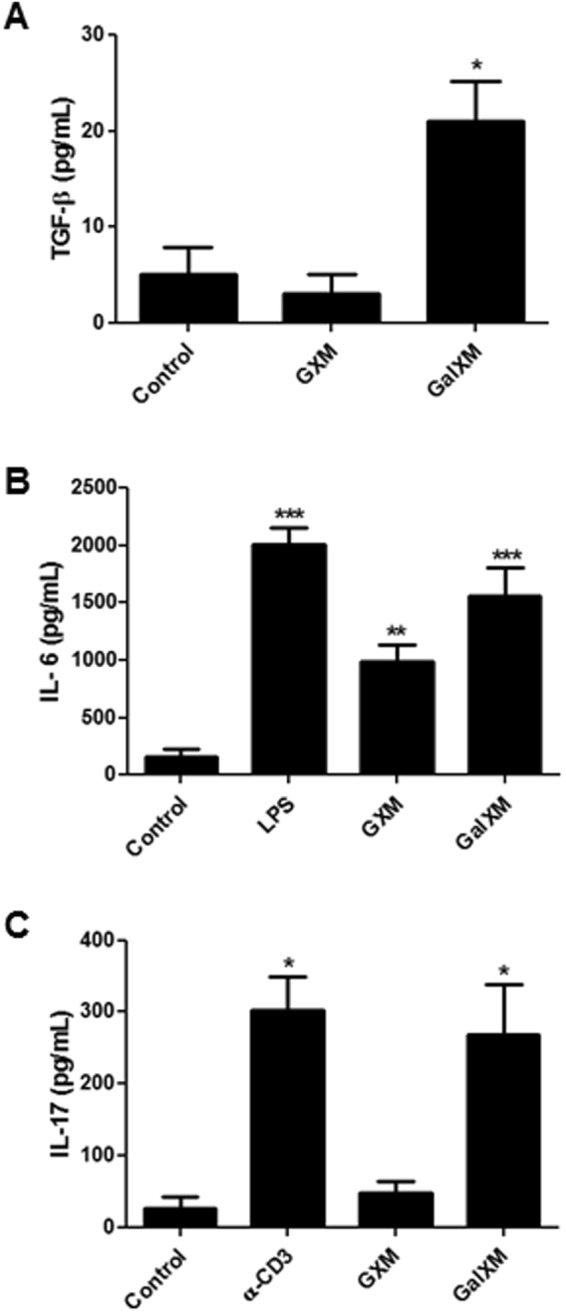
Murine dendritic cells pre-treated with capsular GalXM in co-culture with CD4^+^ T cells induce the production of cytokines that direct the Th17 profile. Dendritic cells (3 × 10^4^/well) were incubated for 24 h in the presence of 50 μg/mL of capsular GXM or GalXM from *C. neoformans*. The dendritic cells were then incubated with CD4^+^ T cells (3 × 10^5^/well) for 48 h. The culture supernatant was collected, and the cytokines (**A**) TGF-β, (**B**) IL-6, and (**C**) IL-17 were analyzed with ELISA assays. The αCD3 and LPS were used at 5 μg/mL and 100 ng/mL, respectively. The control corresponds to dendritic cells without stimulus. The results represent one of four independent experiments. The asterisks indicate ***P < 0.001, **P < 0.01, and *P < 0.05 relative to the control.

A significant question evaluated in this work was the differentiation of T cells to the Th17 subpopulation. The importance of Th17 cells has been well characterized in autoimmune-disease models, and they are important in host defense against various infections caused by bacteria and fungi, particularly on mucosal surfaces^52–57^. Therefore, we evaluated the role of capsular GXM and GalXM in the induction of Th17 cells. In addition to inducing the production of IFN-γ (Fig. 4A), the GalXM-activated dendritic cells surprisingly induced the production of the TGF-β, IL-6, and IL-17 cytokines in the co-cultured CD4^+^ T cells (Fig. 5A,C). We also found that in the presence of GalXM, dendritic cells could secrete IL-23 (Supplementary Fig. S2A), but not IL12p70 (Supplementary Fig. S2B). These results strongly suggest that dendritic cells activated by the GalXM capsular constituent induce the differentiation of CD4^+^ T cells into the Th17 subpopulation.

### Capsular GalXM induces the removal of fungal cells during *C. neoformans* infection

**Figure 6:**
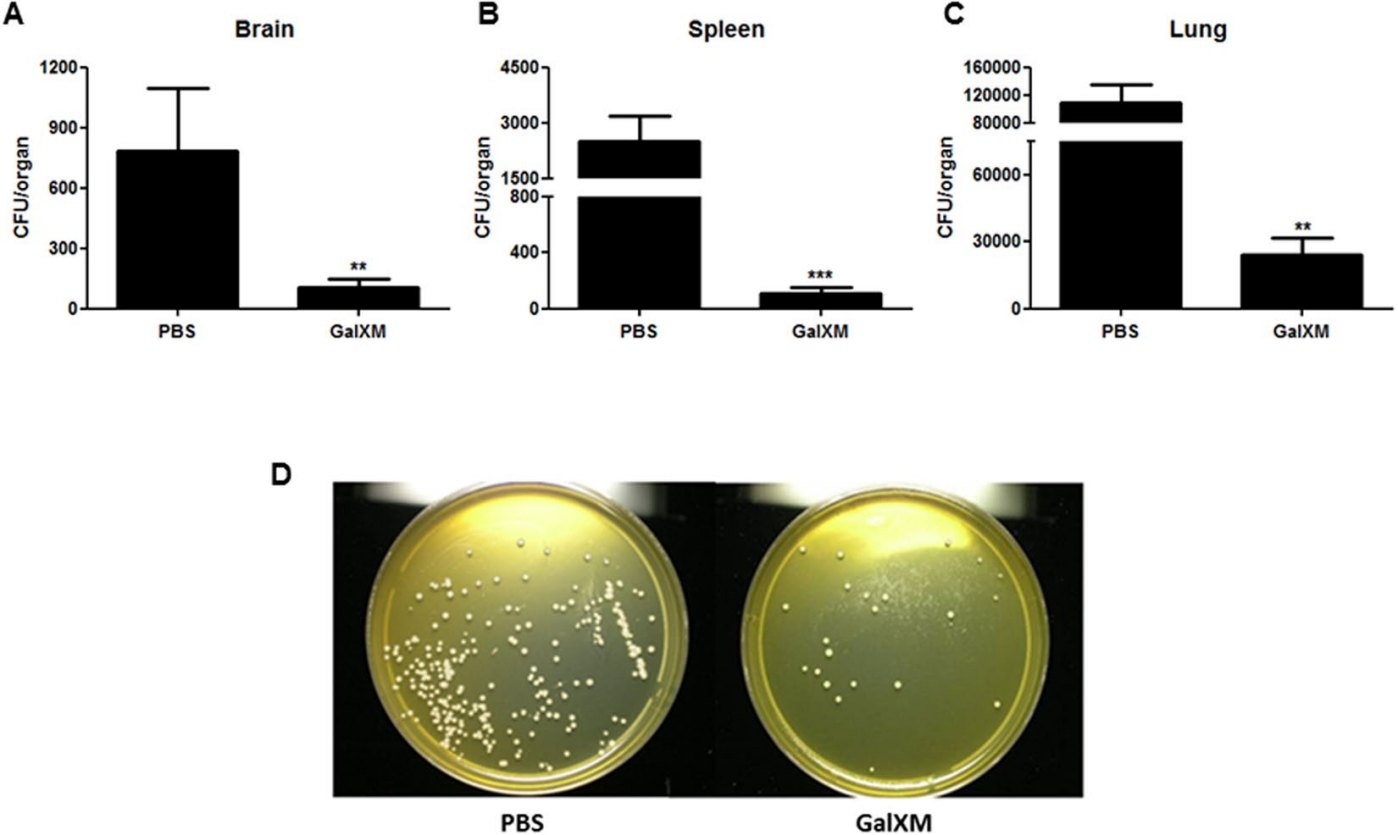
Treatment with GalXM induces the removal of fungal cells from the organs of C57BL/6 mice infected with C. *neoformans*. Mice were pretreated with PBS or capsular GalXM (250 μg/mL intratracheally) 24 h prior to an intratracheal injection with 10^6^ *C. neoformans* cells. After 14 days, the mice were euthanized and the brain, spleen, and lungs were recovered. Viable fungal cells were quantified in petri dishes containing agar from the respective homogenized organs: (**A**) brain, (**B**) spleen, and (**C**) lung. (**D**) Illustrative picture of a lung count. The results are expressed in CFUs (dilutions: 1:2 for brain and spleen; 1:100 for lung). The results represent one of three independent experiments (n = 5 mice/group). The asterisks indicate ***P < 0.001 and **P < 0.01 relative to the control.

The cytokines IL-17 and IL-23 have been described as necessary factors for the protective immune response in murine pulmonary histoplasmosis^58^, and the absence of cytokines IL-6 and granulocyte colony stimulating factor increase susceptibility to *Candida albicans* infection in the murine model^59^. These findings suggest that Th17 effector cells could be involved in immunoprotection during fungal infections. Based on this information, we next evaluated the role of the GalXM during *C. neoformans* infection *in vivo*. Our results showed that treatment with GalXM 24 h prior infection with *C. neoformans* significantly decreased the number of CFUs in the brain (Fig. 6A) and spleen (Fig. 6B) 14 days after infection. In the lung, which is the first site of infection, the number of CFUs was approximately 3-fold lower than that in the control (Fig. 6C). Figure 6D shows that CFUs from the GalXM-treated animals had a similar shape, size, and texture as those from the control. These results demonstrate that capsular GalXM induces the removal of fungal cells in these organs, and the effect remains 30 days after infection (Supplementary Fig. S3). To evaluate whether this decrease in the number of CFUs was a mechanism conserved in other strains of mice, we performed the same infection assay with Balb/c mice. The treatment with capsular GalXM decreased the CFUs in the lung relative to the control 14 days after infection (Supplementary Fig. S4) and in the same way as was seen in the C57BL/6 mice (Fig. 6C).

### IL-17 cytokine is essential for the control of murine cryptococcosis

**Figure 7:**
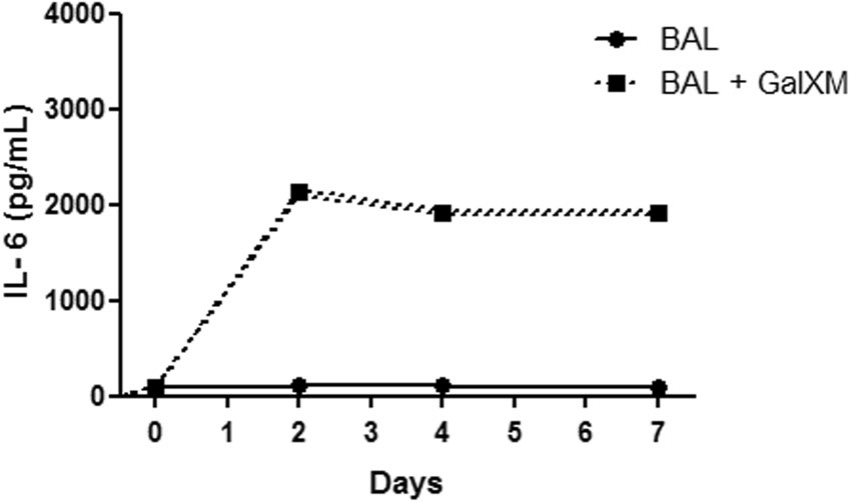
The capsular GalXM constituent induces IL-6 production in bronchoalveolar lavage cells from C57BL/6 mice. Total bronchoalveolar lavage cells (1 × 10^5^/well) were incubated for 24 h before adding 50 μg/mL of capsular GalXM. The culture supernatant was collected at Days 2, 4 and 7 after adding the GalXM, and IL-6 production was determined with ELISA assays. The results represent one of two independent experiments.

To understand the protection mechanisms used by GalXM during the early stages of *C. neoformans* infection in the murine model, we investigated whether the Th17 phenotype was involved in the protection mediated by this capsular constituent during experimental cryptococcosis. Cells belonging to the Th17 subtype produce IL-17, IL-17F, IL-21, and IL-22. The cytokine TGF-β is required to initiate the differentiation process, and IL-6 has been identified as a critical cofactor for Th17 cell differentiation^52,60^. Based on this information and the previously reported modulatory activities mediated by GalXM, we first evaluated the production of IL-6 in a GalXM-stimulated bronchoalveolar lavage. The results confirmed that GalXM induced the production of high concentrations of IL-6 from the second day through the last day of observation (Fig. 7).

**Figure 8:**
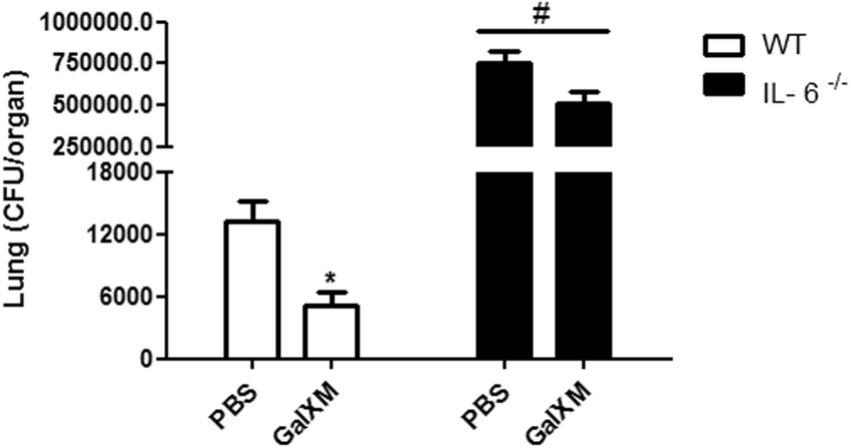
GalXM loses the ability to induce the removal of fungal cells in the lung of *C. neoformans*-infected IL-6^−/−^ mice. Mice were pretreated with PBS or capsular GalXM (250 μg/mL intratracheally) 24 h prior to an intratracheal injection with 10^6^ *C. neoformans* cells. After 30 days, the mice were euthanized, and the lungs were recovered. Viable fungal cells were quantified in petri dishes containing agar from the homogenized organ. The results are expressed in CFUs (dilutions: 1:10 for wild type; 1:1000 for IL-6^−/−^). The results represent one of four independent experiments (n = 5 mice/group). The asterisk indicates *P < 0.05 relative to the control. The hash symbol (#) indicates P < 0.001 compared to the wild-type mice.

**Figure 9:**
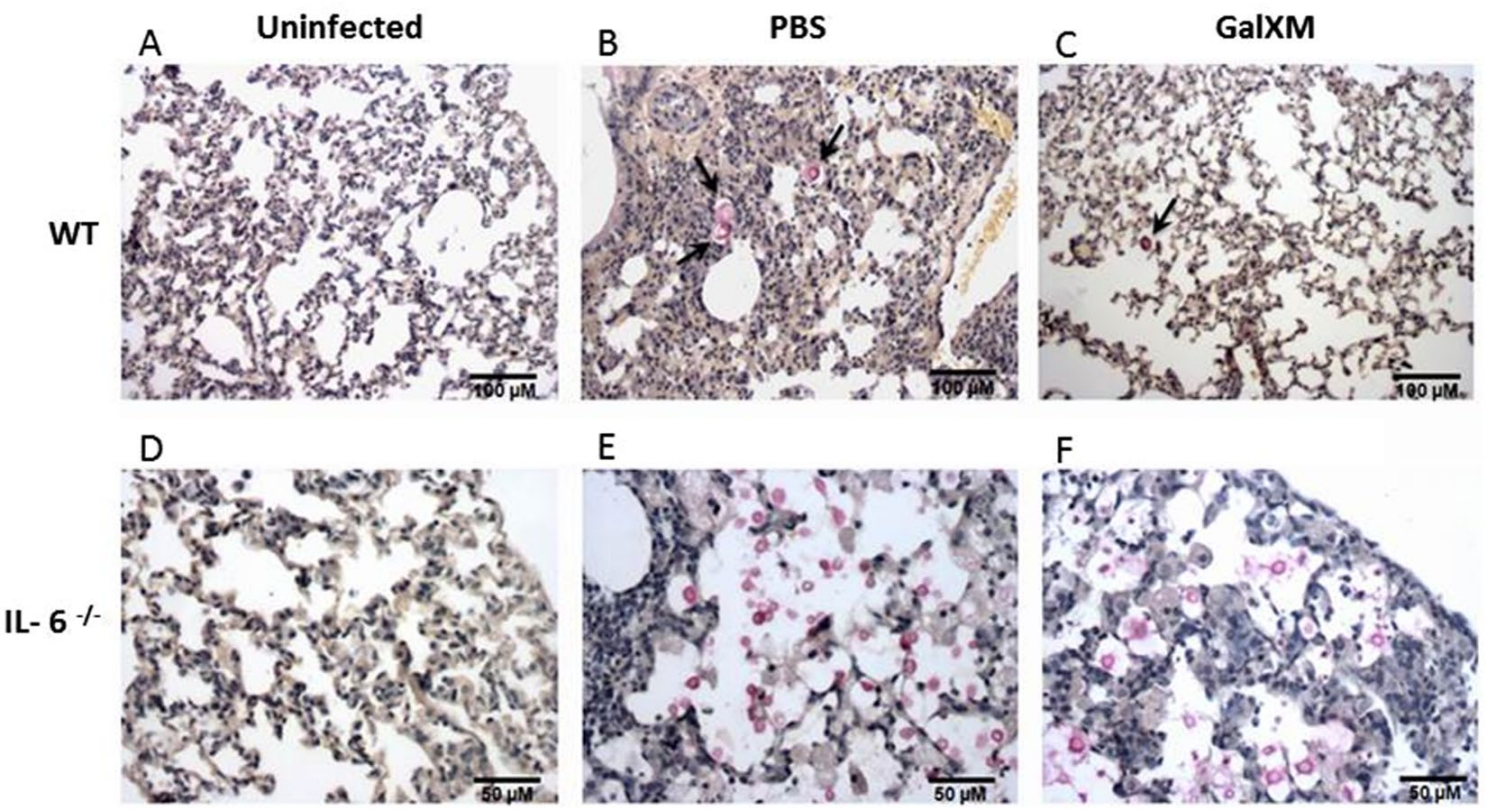
IL-6^−/−^ mice infected with *C. neoformans* produce increased numbers of fungal cells. Interleukin-6 deficient mice (**D–F**) were intratracheally pretreated with PBS (**B,E**) or 250 μg/mL capsular GalXM (**C,F**) 24 h before an intratracheal injection with 10^6^ *C*. *neoformans* cells. After 30 days, the mice were euthanized, and the lungs were recovered. Fungal cells were identified in histological sections positive for mucicarmine. The black arrows in Panels B and C indicate *C. neoformans*. Panels E and F show an abundance of *C. neoformans*. The results represent one of three independent experiments (n = 5 mice/group). The photographs were taken under an optical microscope at 100× (**A–C**) and 200 × (**D–F**) magnification.

**Figure 10:**
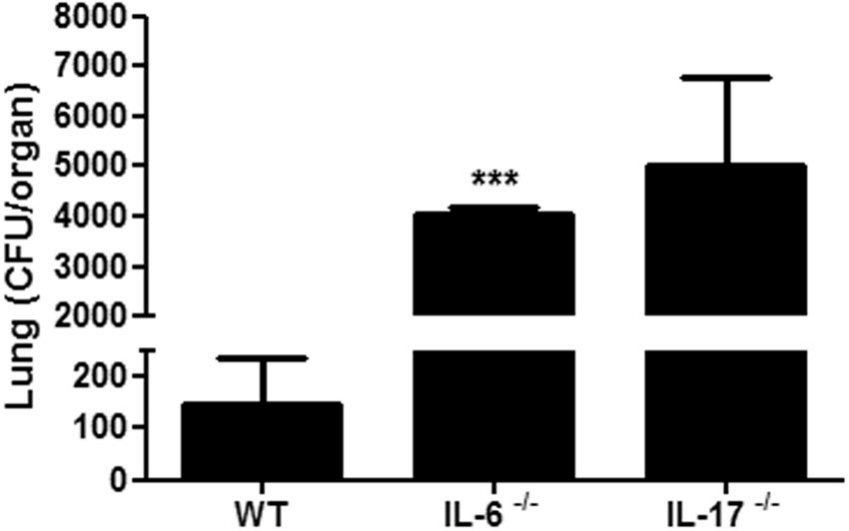
IL-17^−/−^ mice treated with GalXM present a defect similar to that seen in IL6^−/−^ mice in the control of pulmonary infection. Mice (Wt, IL6^−/−^ and IL17^−/−^) were intratracheally pretreated with 250 μg/mL capsular GalXM 24 h before an intratracheal injection with 10^6^ *C. neoformans* cells. After 30 days, the mice were euthanized, and the lungs were recovered. Viable fungal cells were quantified in petri dishes containing agar from the homogenized organ. The results are expressed in CFUs (dilutions: 1:10 for wild type; 1:1000 for IL-6^−/−^ and IL17^−/−^). The results represent one of four independent experiments (n = 5 mice/group). The asterisk indicates *P < 0.05 relative to the control.

We then analyzed the role of IL-6 during *C. neoformans* infection. Our results suggest IL-6 is an essential cytokine for the protective immune response against murine cryptococcosis. In the absence of IL-6 (IL-6^−/−^ mice), the number of CFUs increased approximately 50-fold over the control (Fig. 8). These observations were confirmed by an analysis of lung histological sections (Fig. 9A–F), where a high number of fungal cells were seen in the lung alveoli of the IL-6^−/−^ mice compared to the control animals, suggesting an exacerbation of the infection in the IL-6^−/−^ mice. We also observed important morphological changes in the lungs of the IL-6^−/−^ mice infected with *C. neoformans* relative to the wild-type controls. To evaluate the involvement of GalXM in the IL-17 cytokine-mediated response, we performed the GalXM treatment 24 h before infection and evaluated the number of CFUs in the lungs of animals 30 days after infection. We found that in the absence of IL-6 or IL-17, the capsular constituent GalXM no longer had a protective effect (Fig. 10), suggesting the immunoprotective mechanism of capsular GalXM during a *C. neoformans* infection is Th17 dependent.

## Discussion

In this work, we evaluated the immunomodulatory role of the capsular polysaccharides GXM and GalXM from *C. neoformans* in dendritic cells that are, together with alveolar macrophages, important in regulating the innate immune response against lung infection by *C. neoformans*^61–64^. Initial results using dendritic cells as a model showed that GXM and GalXM induced the increased expression of MHC II and CD86, which are molecules characteristic of mature dendritic cells. In addition, we observed a positive release of cytokine IL-12p40 after the maturation of dendritic cells. The release of IL-12p40 in the presence of different concentrations of the capsule constituents indicated that small concentrations of the two polysaccharides stimulate the dendritic cells to produce IL-12p40, suggesting they contribute to dendritic cell maturation and may induce efficient T-cell responses. These hypotheses are supported by results obtained by Wozniak and colleagues^65^, who showed that dendritic cells isolated from the lungs of mice infected with encapsulated *C. neoformans* had an increase in the expression of the CD80, CD86, and MHC II maturation markers. These results agree with the observation that CD4^+^ T cells in bronchoalveolar infiltrate coincide with the increased expression of two subunits (p40 and p35) of the IL-12 cytokine and with the presence of IFN-γ in the pulmonary homogenate of mice 14 days after infection with *C. neoformans*^66,67^.

It has been shown that acapsular *C. neoformans* is easily phagocytosed by immature dendritic cells, inducing increases in MHC I and II and an increase in the expression of CD40 and CD83^45^. The addition of monoclonal antibodies against GXM facilitated the maturation of dendritic cells, promoting the ingestion of the fungal cells. This maturation allowed for the efficient activation of T cells, their differentiation, and an increase in the production of IFN-γ^45,68^. These authors also investigated the effect of the polysaccharide capsule on genetic expression in dendritic cells by examining the interaction with an acapsular mutant (CAP59) of *C. neoformans*. The results showed that a large number of genes involved in dendritic cell maturation were positively regulated after treatment with the acapsular yeast that was not seen with the capsular cells. However, the author observed a completely different pattern of gene expression in the cells treated with an encapsulated strain^69^. Together, these results suggest that GXM interferes with the activation and maturation of dendritic cells, indicating the fungus may impair the capability for an efficient T-cell response. However, it is important to note that the acapsular yeasts of the CAP59 strain produce GalXM^70,71^ and that these molecules are involved in the maturation of dendritic cells and T-cell activation.

To confirm our hypothesis, we verified the ability of GXM- or GalXM-stimulated dendritic cells to activate T lymphocytes. Our results showed that both the GalXM- and GXM-stimulated dendritic cells were able to induce the proliferation of MLN cells in the presence or absence of ConA. However, unlike the GalXM polysaccharide, GXM was unable to induce the proliferation of MLN cells directly, that is, without the participation of dendritic cells. These data are consistent with the work of Yauch and colleagues^46^, who observed that at high concentrations (300–1000 μg/mL), the GXM capsular component inhibited the proliferation of activated T cells in the presence or absence of dendritic cells. It has also been shown that the inhibition of T cell proliferation was not due to apoptosis or necrosis, but rather due to the inhibition of the IL-2 produced by these cells. This suggests that GXM may have a direct effect on T-cell proliferation in late cryptococcal infections when GXM is present at higher concentrations due to its accumulation in tissues^72^.

The Th1 and Th2 cytokines are involved in infection caused by *C. neoformans*. Although cytokines associated with the Th1 profile are essential for natural immunity, cytokines associated with the Th2 profile do not confer protection in mice^31,73^. It is well described in the literature that the increased expression of Th1 cytokines is related to protection against cryptococcosis through increased phagocytic capacity and macrophage fungicidal activity, resulting in the control of the infection^74–76^. Experiments using IFN-γ^−/−^ mice show an increased fungal load^77^. In addition, an IL-4^−/–^dependent Th2 response exacerbates pulmonary infection^78,79^, and IL-13 (another Th2 cytokine) contributes to fatal allergic inflammation during murine infection with *C. neoformans*^80^.

In our studies, GalXM-stimulated dendritic cells induced the production of IFN-γ, but not IL-4, in CD4^+^ T cells. As already mentioned, GalXM also induces the release of TNF-α in PBMCs, monocytes, and murine macrophages^29,35,81^. These data suggest that GalXM may induce the production of Th1 cytokines in different cell types. However, GXM-stimulated dendritic cells were unable to induce IFN-γ or IL-4 production in CD4^+^ T cells. The GXM capsular polysaccharide has been described as being associated with several immunoregulatory effects and could be considered an immunosuppressive molecule^29,32,82,83^, as its immunosuppressive activity has been well characterized in the literature in recent years^25,29,32,33,83,84^.

Kleinschek and colleagues^85^ showed that the Th17-mediated response is dependent on IL-23 and contributes to protection against infection by *C. neoformans*. In the murine model for *C. neoformans* infection, IL-12 p40- and p35-deficient mice were described as developing a Th2 response associated with high levels of susceptibility^86^. These data strongly suggest that IL-12 plays an essential role in immunity against *C. neoformans*^87^. In addition, IL-17 secretion by splenocytes from *C. neoformans*-infected mice was observed in resistant (IL-13^−/−^) mice, but not in susceptible mice expressing IL-13^80^.

The proliferation of yeast inside macrophages has been characterized as significantly reduced after treatment with the Th1 cytokines IFN-γ, TNF-α, and IL-17^73^. Surprisingly, our results showed that co-cultures of GalXM-stimulated dendritic cells and CD4^+^ T lymphocytes efficiently induced the release of IL-17 and IFN-γ, indicating that this capsular constituent may lead to a Th17 response. These data are supported by Annunziato and colleagues^88^, who found Th17 cells producing IFN-γ in the gut of individuals with Crohn’s disease, which is characterized by a dysregulated Th1/Th17 immune response. Another important study demonstrated that murine *C. neoformans* infection results in the induction of the Th1 response with the release of the IL-17 cytokine, classical macrophage activation, and infection resolution^89^. In 2009, Lin and colleagues^90^ reported that mice vaccinated with Als3p (*Candida albicans* adhesin) for 14 days who then underwent the removal of their splenocytes/lymph nodes and were subsequently re-stimulated with Als3p for 5 days showed an increased production of Th1 (CD4^+^IFN-γ^+^), Th17 (CD4^+^IL-17^+^), and Th1/Th17 (CD4^+^IFN-γ^+^IL-17^+^) cytokines. Moreover, our results showed an increase in the production of cytokines that are essential for the Th17 profile such as the production of TGF-β required for the onset of the phenotype and IL-6, a cytokine characterized as a critical cofactor for the differentiation of Th17^60,91^. We also demonstrated that the production of IL-23 by GalXM-stimulated dendritic cells amplifies and/or stabilizes the Th17 phenotype^52,92^.

To date, we know that the capsular constituent of *C. neoformans* GalXM can induce the maturation of dendritic cells and that these cells can activate T lymphocytes and drive the response to the Th17 phenotype with IFN-γ production, which could be called the Th1/Th17 profile. Some studies have suggested that the Th17 subpopulation, that could be involved in immunoprotection in the fungal-infection model^93^. Recently, Meya and colleagues^94^ have demonstrated that stimulation in patients with *ex vivo* IFN-γ induced a high production of monocytes producing TNF-α and IL-6. Thus, the presence of IFN-γ would favor a differentiation for a Th17 profile. However, a better approach to IFN-γ production is needed in our study model. In addition to the studies of *C. neoformans* infection, the importance of IL-17 has also been described in other fungal-infection models^58,95–97^. Based on this, we decided to examine whether the response to the Th17 subpopulation induced by capsular GalXM-stimulated dendritic cells is related to protection in the murine model during experimental cryptococcosis, despite Zelante and colleagues^98^ having demonstrated that in fungal infections with *C. albicans* and *Aspergillus fumigatus*, an IL-23- and IL-17-mediated response subverted the inflammatory neutrophil program, resulting in severe inflammatory tissue pathology associated with the infections. Surprisingly, our data showed that if treated with GalXM prior to infection, the fungal load was removed, and the effect lasted for the 30-days post infection period that we observed.

The Th1 and Th17 responses resulted in the removal of yeast from the lungs, but they did not prevent the systemic spread of the highly virulent *C. neoformans* strain H99. This suggests that the Th2 response induced by the *C. neoformans* H99 strain is a virulence mechanism in this strain and that the use of Th2 profile-deficient mice (IL-13^−/−^ and IL-4^−/−^) leads to a strong response involving the Th1 and Th17 profiles^76^. However, other mechanisms of virulence could be responsible for the strong central nervous system tropism^76,99^. The mechanisms observed by the authors were not enough to prevent the death of mice that presented with severe pathology in the brain. In our work, we suggested that the capsular polysaccharide GalXM could lead to a protective response mediated by Th1 and Th17 subpopulations, but there are probably other mechanisms involved in preventing the death of mice infected with *C. neoformans* that deserve to be studied. Our results suggest that the capsular polysaccharide GalXM is involved in the protection of mice infected with *C. neoformans*. The data showed that the IL-6 cytokine was essential for the protective immune response against murine cryptococcosis, as demonstrated by the large increase in CFUs in IL-6^−/−^ mice, the high number of fungal cells found within the lung alveoli and the morphological changes in the lung relative to the control. These data suggest that the protective mechanism used by GalXM during *C. neoformans* infection is IL-6 dependent and may involve the Th17 phenotype. The importance of IL-6 during *C. neoformans* infection has also been confirmed in previous work. For example, *C. neoformans* infection was exacerbated in IL-6^−/−^ and IL-12^−/−^ mice, confirming the hypothesis that Th17 and Th1 responses are involved in natural resistance to infection and the induction of a protective response^31,100^.

Administering IL-6 exogenously 24 and 3 hours before intracerebral infection with viable *C. neoformans* cells resulted in a significant reduction in the number of CFUs in the brain and blood, and it increased the survival of infected mice^101^. In addition, PBMCs pre-stimulated *in vitro* for 12 days with heat-killed *C. neoformans* before the addition of viable encapsulated yeasts produced high concentrations of IL-6 compared to PBMCs that were pre-stimulated with feasible fungi^102^. We also showed that the capsular constituent of GalXM induced an increase in IL-6 production by alveolar macrophages and co-cultures of dendritic cells and CD4^+^ T cells.

Finally, we observed the deleterious effect of a lack of the cytokine IL-17^103,104^, which, like IL-6, is important for the resolution and control of *C. neoformans* infection.

It is known that the variations in the extent and position of O-acetylation on the backbone of GXM induces antigenic mutability, which gives rise to the classification of *C. neoformans* strains into five serotypes (A, B, C, D and AD)^105^. Variations related to the capsular polysaccharide GalXM are not yet well characterized. But, it is possible to occur in the constitutions of capsular polysaccharides, and this phenomenon happens mainly by the variation of the culture conditions used and depending on the culture condition used, is a phenomenon often observed in fungi^106,107^. De Jesus *et al*.^108^ demonstrated that differences in sugar composition, physicochemical properties and serological reactivity may occur among GalXM preparations of *C. neoformans* grown under varied culture conditions.

An interesting example mentioned above, the O-acetyl groups are substitutes for polyssacharide capsular GXM and play an important role in the modulation of host immune protectors. For example, the higher virulence observed in *C. gatti* as compared to *C. neoformans* depends on different types of O-acetylation of GXM capsule^109^.

Given the bulky nature of O-acetyl groups and their positioning on the GalXM side chains, we suspect that the extent of O-acetylation may affect immunoregulatory properties of GalXM.

Taken together, our data demonstrated that the capsular polysaccharide GalXM induced maturation of dendritic cells. The presence of this capsular polysaccharide also increased expression of the CD86 and MHC II molecules on the cell surface, followed by IL-23 cytokine production. In addition, GalXM-treated dendritic cells induced T cell proliferation and production of IL-6, IFN-γ and IL-17, suggesting a bias to Th17 response. The properties of GalXM observed in dendritic cells and T cells allowed evaluating their involvement during cryptococcosis. Mice that received GalXM capsular 24 h prior to *C. neoformans* infection were protected as demonstrated by reduced lung, brain and splenic fungal load, suggesting significant involvement of the Th17 subtype in the infection.

## Methods

### Cryptococcus strains

*Cryptococcus neoformans* wild-type (B3501 serotype D) and GXM negative (CAP67 serotype D) strains were kindly provided by Professor Tarnara Doering (Department of Molecular Microbiology, Washington University School of Medicine, St Louis, MO, USA) and Professor Robert Cherniak (Georgia State University, Atlanta, GA, USA), respectively. The cells were cultured in a liquid defined medium (LDM)^110^ at 28 °C −30 °C with continuous shaking (100 rpm) for 5 days for polysaccharides purification and 2 days for infection experiments before removal by centrifugation.

### Ethics statement

This study was carried out in strict accordance with the recommendations in the Guide for the Care and Use of Laboratory Animals of the National Institutes of Health (USA). The protocol was approved by the Committee on the Ethics of Animal Experiments of the Health Science Center of the Federal University of Rio de Janeiro (CEUA-CCS, Permit Number: A17/17-061-14) and all efforts were made to minimize suffering.

### Mice

Six to eight week-old Balb/c, C57BL/6, C57BL/6 IL-6^−/−^ (IL-6 deficient), and C57BL/6 IL-17^−/−^ (IL-17 deficient) mice were purchased from the University of São Paulo. The mice were kept in polypropylene boxes in a temperature-controlled environment between 23 °C and 25 °C and had free access to water and standard feed during the experiments.

### Isolation and purification of *C. neoformans* capsular polysaccharides (GalXM and GXM)

Isolation and purification procedures were adapted from protocols mentioned before^19,29^. The GalXM was purified from culture supernatant from *C. neoformans* CAP67 mutant strain grown as described above. Supernatants were separated from the cells by centrifugation at 1200 × *g* for 30 min at 4 °C, concentrated by ultrafiltration using a 10 kDa-cutoff spiral cartridge (Amicon/Millipore, USA), and submitted to overnight precipitation at 4 °C after addition of three volumes of ethanol. The precipitate containing GalXM and mannoprotein was dissolved in distilled water, filtered through a 0.45 μm filter and lyophilized. The freeze-dried mixture of GalXM and mannoprotein was dissolved in 10 mL of 5 mM sodium acetate buffer pH 5.2, containing 1 mM calcium chloride, 1 mM magnesium chloride, 1 mM manganese chloride and 0.15 M sodium chloride (Con-A buffer), and applied to a XK-26/40 column packed with 70 mL ConA-Sepharose 4B (GE Healthcare, Sweeden), equilibrated in the same buffer and connected to an HPLC system (AKTApurifier GE Healthcare, Sweeden) at a flow rate of 0.25 mL/min. The column was washed with 5 column volumes (CV) of Con-A buffer to elute the GalXM. The flow through and washes were collected as fractions of 10 mL and assayed with phenol-sulfuric acid reaction^111^. Fractions containing GalXM were pooled, concentrated, dialyzed against distilled water and lyophilized.

The GXM capsular polysaccharide was isolated from *C. neoformans* B3501 wild type strain and purified by differential precipitation with CTAB^12^. Briefly, capsular polysaccharides isolated from the culture supernatant by precipitation with ethanol were dissolved in 0.2 M NaCl, CTAB (3 mg/mg of polysaccharides) was added slowly. Then, a solution of 0.05% CTAB was added and the GXM was selectively precipitated. The precipitate was collected by centrifugation and washed with 2% acetic acid in ethanol then in 90% ethanol. The precipitate was dissolved in 1 M NaCl and three volumes of ethanol were added to precipitate the GXM. The precipitate was centrifuged, washed with 2% acetic acid in ethanol and then with ethanol, dissolved in water and lyophilized.

To eliminate potential LPS contamination, 10 mg of GalXM or GXM preparations were dissolved in LPS-free water and further purified through chromatography on a column of Polymyxin B-Agarose (Sigma) equilibrated with LPS free-water. LPS-free GalXM or GXM were eluted with 12 mL of water, recovered and lyophilized.

### Intratracheal infection

The animals were injected intratracheally with 250 μg/mL GalXM or PBS. After 24 h, the animals were intratracheally infected with the encapsulated yeast strain *C. neoformans* (strain B3501 serotype D). Through the hemocytometer count, an inoculum containing 10^6^ viable cells (or 5 × 10^6^ for the survival assay) was made and observed for 14 (C57BL/6 and Balb/c) or 30 (C57BL/6, IL-17^−/−^, and IL-6^−/−^) days.

### Dendritic cells

Dendritic cells were obtained as described by Lutz and colleagues^112^. The tibia and femur of C57BL/6 mice were dissected and the marrow removed using a syringe containing RPMI medium. The cells were homogenized, counted, and adjusted to 2 × 10^6^ cells/plate in RPMI medium supplemented with 2 mM L-glutamine, 5 × 10^−5^ M β-mercaptoethanol, 1 mM sodium pyruvate, 1 mM non-essential amino acids, 10% SFB, and 20 ng/mL rmGM-CSF (recombinant granulocyte macrophage mouse growth factor; R&D Systems). A 10-mL suspension was transferred to a petri dish and incubated at 37 °C in a 5% CO_2_ atmosphere for 10 days. Fresh rmGM-CSF (20 ng/mL) was added every third day of culture.

### Syngeneic lymphocyte reaction

Differentiated C57BL/6 dendritic cells (5 × 10^5^/well) were incubated with 50 μg/mL GXM or GalXM (isolated from the wild-type B3501 strain) in supplemented RPMI medium containing 10% SFB for 24 h at 37 °C in a 5% CO_2_ atmosphere. The cells were then removed, washed, and re-incubated (3 × 10^4^/well) in microplates with purified CD4^+^ T cells (3 × 10^5^/well) for another 48 h.

### Lymphoproliferative response

Differentiated dendritic cells (5 × 10^5^/well) from C57BL/6 mice were incubated with 50 μg/mL GXM or GalXM for 24 h. The cells were then removed, washed, and re-incubated (3 × 10^4^ cells/well) with mesenteric lymph node (MLN) cells (3 × 10^5^/well) with or without ConA (5 μg/mL) for 72 h. For the direct lymphoproliferative response assay, we used MLN cells (3 × 10^5^/well) incubated with 50-μg/mL GXM or GalXM^29^ in the presence of anti-CD3 (5 μg/mL) for 72 h. After this period, 10 μL of a 1:10 dilution of a 5 mCi (185 MBq) ^3^H-thymidine stock solution was added to the culture. The incorporation of ^3^H-thymidine was determined by liquid scintillation (LS6500 Beckman Coulter scintillation counter) after further incubation for 18 h.

### Cytokine assay

Dendritic cells from C57BL/6 mice (2 × 10^5^/well) were incubated for 24 h with 50 μg/mL of GXM derived from *C. neoforman*s. The supernatants were then collected, and enzyme-linked immunosorbant assays (ELISAs) were used to determine the production of the cytokines IL-4, IFN-γ, IL-12p40, IL-10, IL-23, IL-12p70, IL-17, IL-6, TNF-α, and TGF-β. We also used an ELISA assay (BD Biosciences) to quantify the production of IL-6 in total bronchoalveolar lavage cells.

### Histopathological studies

The mice were euthanized after 30 days of infection, and the lungs, brain, and spleen were removed and fixed in 10% buffered formalin for 24 h. The organs were embedded in paraffin, sliced (5–6 μm thick) with a Leica microtome, and stained with the mucicarmine technique. The stained sections were analyzed under a Nikon Eclipse E400 microscope.

### Colony forming units (CFUs)

The lungs, brains, and spleens were macerated in 1-mL sterile PBS and 10-fold serial dilutions were plated in duplicate on Sabouraud dextrose-agar plates. Neat and 1:10 dilutions of freshly extracted blood were similarly plated. The plates were allowed to grow for 72 h at 30 °C before counting the visible colonies to determine the total CFUs per organ.

### Statistical analysis

All calculations were performed using GraphPad Prism v5.0. Results in the figures are expressed as the mean and standard deviation (SD). The number (*N*) of animals per group is indicated in the figure legends. Data were compared by analyses of variance (ANOVA) followed by a Dunnett or Tukey post-test. Significant differences are indicated for *P < 0.05 **P < 0.01, and ***P < 0.001.

## Electronic supplementary material

Supplementary Information
