# Supplementary material for: Involvement of the capsular GalXM-induced IL-17 cytokine in the control of Cryptococcus neoformans infection
Source: Sci Rep. 2018 Nov 6;8:16378. doi: 10.1038/s41598-018-34649-4 (PMC6219535; doi:10.1038/s41598-018-34649-4)
Supplement: Supplementary file 1 — Supplementary Information [file 41598_2018_34649_MOESM1_ESM.doc]

# SUPPLEMENTARY INFORMATION

**Involvement of the capsular GalXM-induced IL-17 cytokine in the control of *Cryptococcus neoformans* infection**

*Isabel Ferreira LaRocque-de-Freitas^1^, Juliana Dutra B. Rocha^1^, Marise Pinheiro Nunes^2^, Priscila Angelica V. Oliveira^1^, Danielle de Oliveira Nascimento^1^, Leonardo Freire-de-Lima^1^, Christina Maeda Takiya^1^, Alexandre Morrot^2,3^, Debora Decote-Ricardo^4^, Jose Osvaldo Previato^1^, George A. DosReis^1^, Lucia Mendonça-Previato^1*^, Celio Geraldo Freire-de-Lima^1*^*

^1^Instituto de Biofísica Carlos Chagas Filho, Universidade Federal do Rio de Janeiro, Rio de Janeiro, Brasil.

^2^Laboratorio de Imunoparasitologia, Instituto Oswaldo Cruz, FIOCRUZ, Rio de Janeiro, Brasil.

^3^Faculdade de Medicina, Universidade Federal do Rio de Janeiro, Rio de Janeiro, Brasil.

^4^Instituto de Veterinária, Universidade Federal Rural do Rio de Janeiro, Seropédica, Brasil.

Correspondence and request for materials should be addressed to C.G.F-de-L (celio@biof.ufrj.br) or L.M-P. (luciamp@biof.ufrj.br).


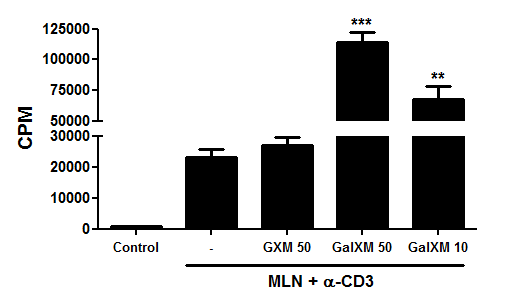


**Supplementary Figure S1. Lymphoproliferative response induced by the capsular constituent GalXM.** Mesenteric lymph node (MLN) cells (3×10^5^/well) were incubated for 72 h in the presence of different concentrations (μg/mL) of the capsular constituents from *C. neoformans*. Lymphocyte proliferation was analyzed by the incorporation of 3H-thymidine in the last 18 hours of culture. Anti-CD3 was used at a concentration of 5μg/mL. The control corresponds to MLN cells without anti-CD3 and without stimulus. The results represent one of three independent experiments. Asterisks indicate ***P <0.001 and **P <0.01 relative to MLN cells with anti-CD3, but without stimulation.


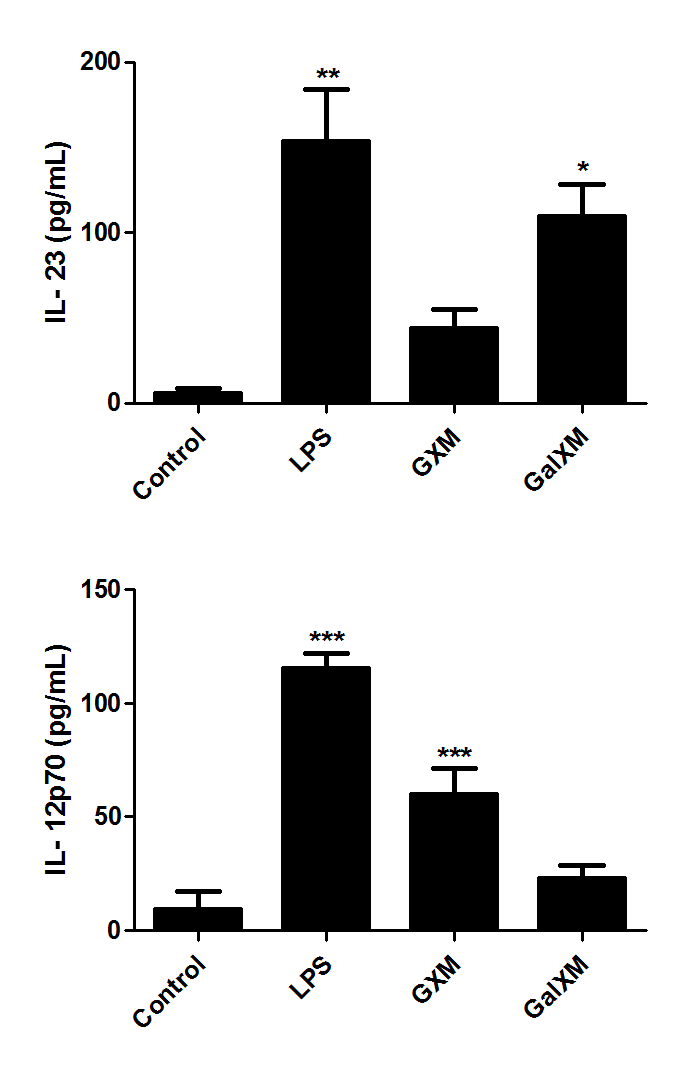


**A)**

**B)**

**Supplementary Figure S2. Treatment with GalXM induces the production of IL-23 by dendritic cells.** Dendritic cells (2×10^5^ cells/well) were incubated for 24 h with 50 μg/mL of the capsular polysaccharides GXM or GalXM from *C. neoformans*. The culture supernatant was collected, and the production of IL-23 and IL-12p70 was analyzed with ELISA assays. The control (100 ng/mL LPS) corresponds to dendritic cells without stimulus. The results represent one of three independent experiments. The asterisks indicate ***P <0.001, **P <0.01, and *P <0.05 relative to the control.


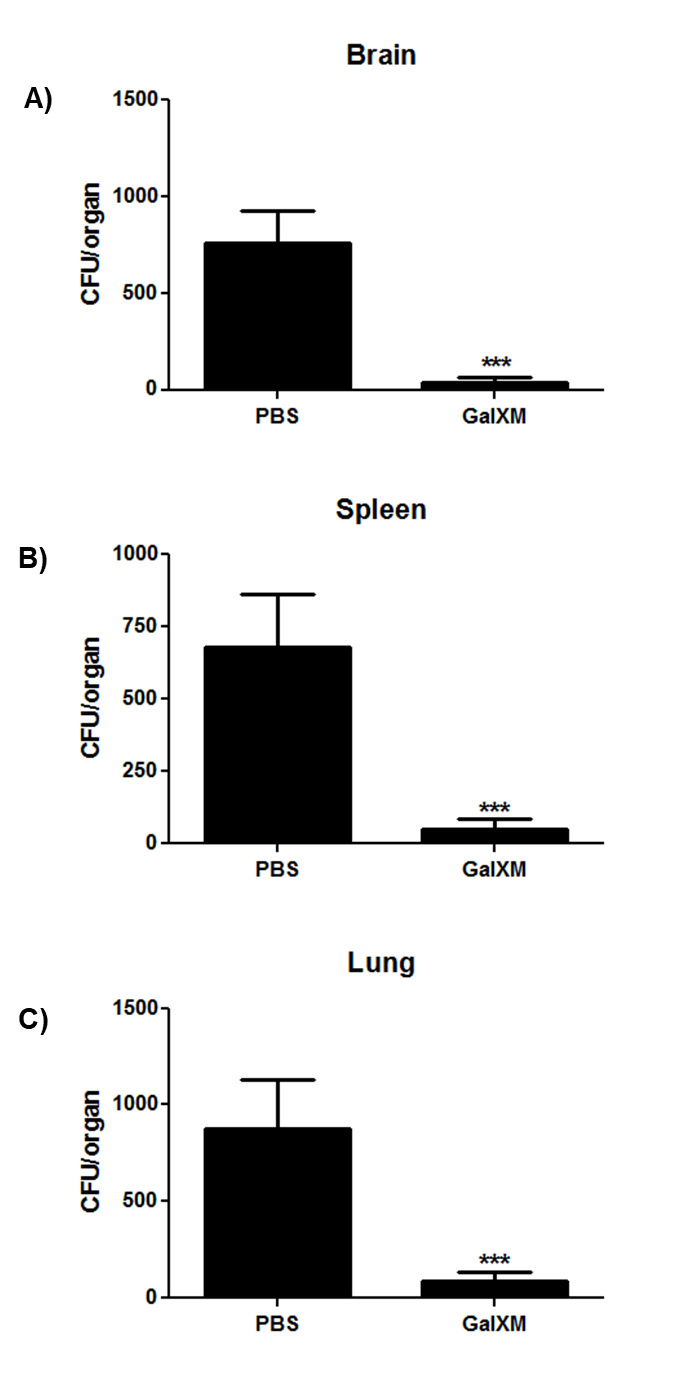

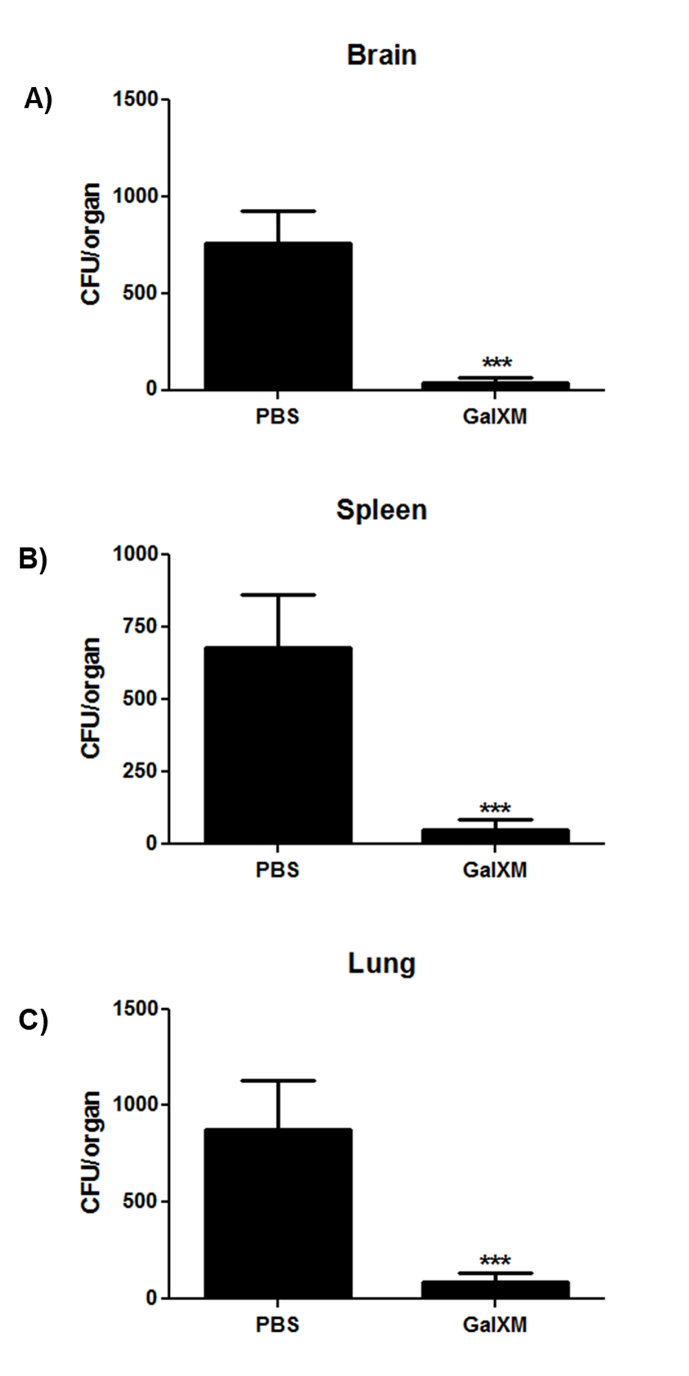


**Supplementary Figure S3. Effect of GalXM-induced removal of fungal cells on the organs of *C. neoformans*-infected C57BL/6 mice remains after 30 days of infection.** Mice were pretreated with PBS or capsular GalXM (250 μg/mL intratracheally) 24 h prior to an intratracheal injection with 10^6^ *C. neoformans* cells. After 30 days, the mice were euthanized and the (A) brain (B) spleen, and (C) lungs were recovered. Viable fungal cells were quantified in petri dishes containing agar with the respective homogenized organ. The results are expressed in CFUs (dilutions: 1:2 for brain and spleen; 1:10 for lung). The results represent one of three independent experiments (n = 5). The asterisks indicate ***P <0.001 and **P <0.01 relative to the control.


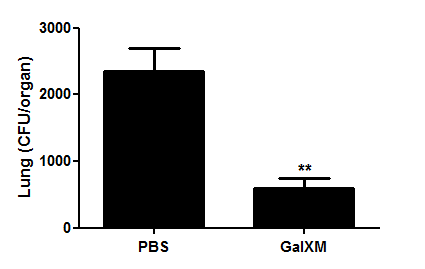


**Supplementary Figure S4. Treatment with GalXM induces the removal of fungal cells in the lungs of *C. neoformans*-infected Balb/c mice.** Mice were pretreated with PBS or capsular GalXM (250 μg/mL intratracheally) 24 h prior to an intratracheal injection with 10^6^ *C. neoformans* cells. After 14 days, the mice were euthanized, and the lungs were recovered. Viable fungal cells were quantified in petri dishes containing agar from the homogenized organ. The results are expressed in CFUs (1:100 dilution). The results represent one of two independent experiments (n = 5). The asterisks indicate **P <0.01 relative to the control.
